# Sensory stimulations potentializing digital therapeutics pain control

**DOI:** 10.3389/fpain.2023.1168377

**Published:** 2023-09-06

**Authors:** Maxime Fougère, Juliette Greco-Vuilloud, Chloé Arnous, Florence Abel, Chrissy Lowe, Valery Elie, Serge Marchand

**Affiliations:** ^1^ Lucine, Bordeaux, France; ^2^ Faculté de Médecine et des Sciences de la Santé, Centre de Recherche Clinique du Centre Hospitalier Universitaire de Sherbrooke, Université de Sherbrooke, Sherbrooke, QC, Canada

**Keywords:** digital therapeutics, pain, virtual reality, analgesia, hypnosis, binaural beats, colored noise, bilateral alternative stimulation

## Abstract

For the past two decades, using Digital Therapeutics (DTx) to counter painful symptoms has emerged as a novel pain relief strategy. Several studies report that DTx significantly diminish pain while compensating for the limitations of pharmacological analgesics (e.g., addiction, side effects). Virtual reality (VR) is a major component of the most effective DTx for pain reduction. Notably, various stimuli (e.g., auditory, visual) appear to be frequently associated with VR in DTx. This review aims to compare the hypoalgesic power of specific stimuli with or without a VR environment. First, this review will briefly describe VR technology and known elements related to its hypoalgesic effect. Second, it will non-exhaustively list various stimuli known to have a hypoalgesic effect on pain independent of the immersive environment. Finally, this review will focus on studies that investigate a possible potentialized effect on pain reduction of these stimuli in a VR environment.

## 1. Virtual reality

Many virtual reality (VR) definitions have been proposed in the past few decades, from short straightforward to more complex explanations. Honzel et al. elegantly summarized it as follows: an immersive computer-generated environment designed to be perceived as real by the user (56). Meanwhile, Digital Therapeutics (DTx) has been defined as an “*evidence-based therapeutic interventions that are driven by high-quality software to treat, manage, or prevent a disease or disorder* […] *used independently or in concert with medications, devices, or other therapies to optimize patient care and health outcomes*” (138). Interestingly, DTx benefit from VR technologies in the healthcare system (21), particularly since the COVID-19 pandemic, which led to a more digitalized model (22). Thus, VR has been increasingly studied, notably in acute or chronic pain analgesia situations (128).

The hypoalgesic power of VR has been extensively highlighted in recent meta-analysis and reviews not only in the adult population (1, 9, 16, 17, 25, 47, 72, 76, 78, 102) but also in pediatric patients (28). In addition, benefits affecting several modalities of quality of life have been reported (e.g., stress, anxiety), suggesting VR as a good non-pharmacological therapeutic tool (95, 76, 150). The goal of this review is to verify if the addition of different auditory and visual stimulations frequencies [e.g., binaural beats (BBs), hypnosis] have additive effects on VR efficacy. The first part of this review will exclusively focus on pain studies that investigated the hypoalgesic effects of VR in acute or chronic pain conditions, followed by the physiological evidence supporting this effect. The second part will non-exhaustively list several sensory stimuli used to promote analgesia as stand-alone treatments. Finally, the third part of this review will aim to investigate studies combining one or many of the sensory stimuli previously described in a VR environment (Table 1).

**Table 1:** List of the articles that refer to the different stimulations with and without VR for acute and chronic pain.

Stimulation modalities		Acute pain	Chronic pain
VR		(2, 3, 5, 7, 15, 38, 45, 50, 51, 53, 54, 55, 118)	(10, 40, 63, 65, 92, 97, 113, 120, 135, 148)
Sensory stimuli alone	Hypnosis	(32, 33, 35; 60, 62, 142)	(127)
	Binaural beats	(6, 27, 87, 98, 101)	(151)
	Colored noise	(11, 67, 71)	(12, 41)
	Bilateral alternative stimulation	(48, 79)	(42, 44, 80)
VR + sensory stimuli	VR + Hypnosis	(20, 110, 111)	(96)
	VR + Binaural beats	(N/A)	(99, 100, 109)
	VR + Collared noise	(64)	(N/A)
	VR + Bilateral alternative stimulation	(66)	(66)

### 1.1. Analgesic power of virtual reality

#### 1.1.1. Acute pain

Pain is a perception mechanism aiming to alert the organism of nociceptive stimuli potentially compromising its survival. Since 2020, its definition has been revised by the International Association for the Study of Pain (IASP) as follows: “*An unpleasant sensory and emotional experience associated with, or resembling that associated with, actual or potential tissue damage*” (106).

**Figure 1:**
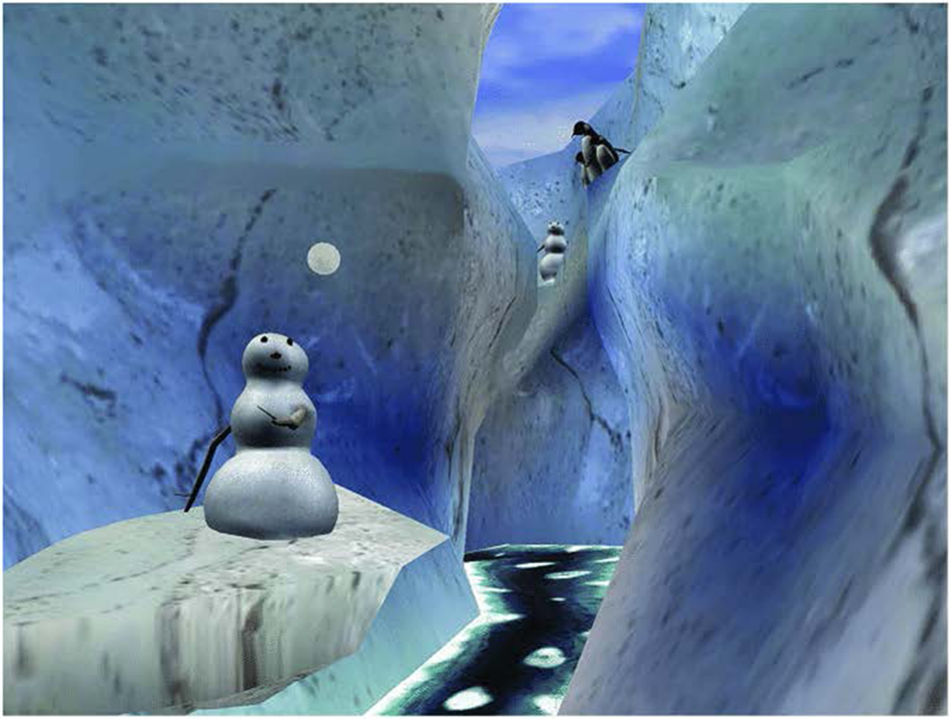
SnowWorld, a 3D virtual reality environment designed in the early 2000s for analgesia purposes in burnt victims [adapted from Honzel et al. (56)].

To our knowledge, the first evidence of VR analgesia on acute pain came from the work of Hoffman et al. in the early 2000s. First, they reported a decrease of the perceived pain following a VR session in two adolescent patients during burn wound care (Figure 1) (50). Second, using magnetic resonance imaging (MRI), they highlighted that VR effectively lowered brain activity in areas related to pain (i.e., anterior cingulate cortex, primary and secondary cortex, insula, and thalamus) in 14 healthy participants (51). Third, they reported the importance of choosing a good quality VR headset to improve the efficiency of the device (52). Fourth, they have shown that VR significantly reduces pain compared to opioids, with a potentialized effect when both are being coupled (53). Following these results, they continued to provide significant evidence concerning the hypoalgesic power of VR through pain measurements and cerebral imaging, mainly in burn victims or children receiving painful procedures (2, 3, 5, 7, 54, 55). Following the release of these pioneer studies performed by Hoffman et al., many teams have now shown a hypoalgesic effect of VR in acute pain situations, such as venipuncture, lumbar puncture, women during labor, or dental surgery (15, 45, 38, 118).

In accordance with these results, recent reviews are increasingly reporting the benefits of VR for acute pain analgesia (1, 25, 47, 58, 72, 76, 78, 102). For instance, it has been shown that VR is able to significantly increase thermal pain tolerance (46) or significantly decrease experimental pain intensity using electrical- and thermal-induced stimuli (119). Moreover, it has been highlighted that three sessions a day of 30 min of VR is also efficient to reduce pain during a rehabilitation protocol after knee surgery (63). Interestingly, the addition of VR with local anesthesia in patients undergoing dental surgery can significantly reduce oxygen saturation, intraoperative pulse rate, and postoperative visual analog pain scale results (126).

However, it is important to notice that some studies did not find significant results regarding a hypoalgesic effect of VR on some acute pain stimulation. Indeed, Walker et al. investigated the efficacy of VR distraction during a cystoscopy, without a significant decrease of pain questionnaire scores between the VR and control groups (145). The authors suggested that these results could likely be due to a lack of immersion, which is the main mechanism of action during a VR experience (see Section 1.2). In addition, Smith et al. tested the pressure pain during various contextual conditions and did not show statistical differences on participants’ pain thresholds (121). However, the authors mentioned that they investigated pain *sensitivity* instead of pain *intensity*, which could explain their results. Importantly, these disparate results also highlight the difficulty for VR studies to properly choose the best methods (e.g., protocol of administration, VR apparatus), thus leading to difficulties to compare the results between studies.

Interestingly, the VR hypoalgesic effects observed on acute pain are not limited to the duration of the application. It has been reported that this effect can last up to 1 h post-VR application following either a cardiac surgical or an episiotomy repair procedure (59, 86). Further investigations are required to evaluate this lasting hypoalgesic effect of VR on acute pain (Table 1).

#### 1.1.2. Chronic pain

Chronic pain can be defined as a persistent or recurrent pain, lasting for at least 3 months (136, 137). Chronic pain can lead to critical dysfunctions in both peripheral and central nervous systems, such as gray and white matter loss, increase or decrease of the activity in major cerebral areas, or alterations of synaptic neurotransmission (124, 108). In addition, chronic pain may severely affect the quality of life of patients living with it, from nutrition and physical activity to sleep disorders and mental wellbeing (91, 85). Importantly, chronic pain can appear through aging and in patients presenting specific diseases (77).

While current results are converging toward the efficacy of VR to diminish acute pain, reviews are also looking to the potential hypoalgesic power of VR on chronic pain (47, 78, 102). Interestingly, major significant VR benefits on pain ratings or pain relief have been reported in various chronic conditions, such as musculoskeletal pain, neuropathic pain, cervical/thoracic/lumbar spine pain, hip pain, pervasive pain, or interstitial cystitis (63, 65).

A study on six fibromyalgia patients has shown that pain reduction was significantly greater in the VR group compared to the control group (10). Another study has highlighted that VR immersion can significantly reduce pain perceived in patients living with chronic migraines in a hospital waiting room (135). Similarly, VR benefits have been found in children with chronic headaches (120). Another one has shown a major decrease of pain intensity in patients suffering from chronic pain (148). Also, an improvement of pain intensity, frequency, duration, and intrusion in patients living with phantom limb pain after 12 VR sessions has been reported (92). In addition, pain intensity was significantly decreased after a 6-week VR session in patients with subacromial impingement syndrome (97). Recently, a 56-day study has highlighted a significant decrease of pain intensity in patients living with chronic low back pain (40).

The duration of the hypoalgesic effect of VR on chronic pain varies across the studies (78). The benefits have been demonstrated to last up to (i) 1 month (97); (ii) 1 to 3 months (120, 135); (iii) 1, 3, and 6 months (92); or even (iv) 6 months (10) post-treatment, potentially suggesting a long-term efficacy of VR on chronic pain. Finally, it is important to notice that some studies did not find significant results regarding the hypoalgesic effects of VR, or at least sustainable ones (113), on unspecified chronic pain conditions (63, 148).

In conclusion, ample evidence attests to the many benefits of VR on acute and chronic pain analgesia, while some pointed out the lack of an effect. Nevertheless, the study of this technology in these pain conditions is still under investigation in various pathologies, allowing us to better understand the limits of its hypoalgesic power (Table 1).

### 1.2. Physiological evidence supporting the analgesic efficacy of VR

The exact mechanisms through which VR is procuring the hypoalgesic effects previously mentioned on acute and chronic pain are still unclear, although it seems that the main one is immersion (18, 74). This concept needs to be differentiated from distraction. Indeed, it has recently been shown that immersive VR significantly increases heat-pain tolerance limits, as well as improves mood, situation anxiety, and pain unpleasantness, while a distraction control only increased the pain tolerance limits without affecting the other modalities (18). In addition, the authors highlighted that the increase of the heat-pain tolerance limits by VR was related to an increase of sympathetic and parasympathetic responses (e.g., heart rate variability standard deviation from normal to normal, galvanic skin responses). Interestingly, it has also been shown that VR cues related to “virtual water temperature” (i.e., color red for hot and blue for cold) can significantly influence the pain perception of thermal stimuli (68). Using the same nociceptive thermal stimulation, a virtual hot water signal was perceived as significantly more painful than a virtual cold signal, likely through top-down endogenous mechanisms.

This hypoalgesic ability of VR leads us to compare it to traditional medication. To date, the most common analgesics prescribed for pain are opioids (89). Notably, repetitive opioid use can lead to an increase of postoperative acute pain episodes (37) as well as major aversive effects, such as addiction or overdose death (112). Thus, the current worldwide opioid crisis has led to an urgency to find new non-pharmacological hypoalgesic solutions, in which VR appears to be effective (132). As previously mentioned, VR can be as efficient as opioids to reduce pain, with a potentialized efficiency when used adjunctively (53). In addition, VR can effectively reduce opioid administration during painful wound care procedures (82). Thus, the current emergence of various DTx using VR is very interesting in terms of novel hypoalgesic strategies.

One of the main counter-indications of VR is the adverse effect known as “cybersickness,” mainly causing nausea symptoms in VR users (70, 81, 144, 146). It seems that this motion sickness could be due to a conflict between the sensory stimuli or autonomic responses (e.g., visual system, vestibular system) (70). Interestingly, a recent review highlighted a close relationship between cybersickness and the feeling of presence in a VR environment: the more the cybersickness experienced, the less immersed the VR user will be (146), which could prevent any hypoalgesic effects of DTx using VR.

Several sensory (i.e., auditory, visual, olfactory, gustatory, tactile) stimuli can be transmitted to users through a VR apparatus in order to increase immersion within the virtual environment (1, 68), allowing the transmission of various stimulations [e.g., hypnosis, binaural beats, colored noise, bilateral alternative stimulation (BAS)], thus potentially being able to increase the VR hypoalgesic power.

## 2. Sensory stimuli

Among the various sensory stimuli that DTx may use to induce a hypoalgesic effect, VR technologies for pain treatment frequently include therapeutic scripts as well as sound and light frequencies in the virtual environment (83). It is difficult to separate the efficacy of VR from the effects of some visual or auditory stimuli since they are presented together in most of the studies. We will introduce a non-exhaustive description of psychological (i.e., hypnosis), auditory (i.e., binaural beats, colored noise), and visual (i.e., bilateral alternative stimulation) stimuli that are the most used in VR, but can be used as a stand-alone treatment (Table 1).

### 2.1. Hypnosis

The definition of hypnosis has frequently evolved throughout the years, mainly to increase its comprehension, in order to allow its use, notably, in scientific studies (29). One of the more recent is the following: “*A state of consciousness involving focused attention and reduced peripheral awareness characterized by an enhanced capacity for response to suggestions*” (29). Several clinical studies have highlighted the benefits of hypnosis in several situations (e.g., pain, depression, motor paralysis, phobia) (8, 103, 105, 129, 142).

Analgesia through hypnosis has majorly been investigated during surgeries for “hypnosedation” (35, 69, 142). A pioneer retrospective study has shown that adding hypnosedation to a conscious intravenous sedation coupled with a local anesthesia improves both perioperative pain and anxiety relief during various plastic surgery (e.g., breast augmentation, correction of mammary ptosis, liposuction) compared to medical sedation alone (32). These results were confirmed a few years later during a prospective study (33). More recently, a critical review of nearly 30 randomized controlled clinical trials (RCTs) highlighted that hypnosis was consistently able to decrease pain in acute painful conditions compared to standard care and attention control groups (69). The authors also mentioned that hypnosis was at least as efficient to comparable adjunct psychological or behavioral therapies in the same context. Importantly, it has been shown that hypnosis was able to significantly reduce pain intensity in patients suffering from chronic pain following a spinal cord injury, compared to non-invasive electrical stimulation (60, 62). Hypnosis has been reported to more effectively reduce the pain intensity in chronic low back pain participants using self-hypnosis training compared to biofeedback (127). Finally, a recent review and meta-analysis reported that hypnosis was moderately able to manage pain in musculoskeletal and neuropathic pain patients, suggesting that deeper investigations were necessary to conclude on the hypoalgesic power of hypnosis on chronic pain conditions (73).

In addition to its hypoalgesic power, hypnosis can effectively impact the patient's quality of life, as well as multiple medical spheres (e.g., pain, medication consumption, physiological parameters, recovery and professional activity restart latency, emotional distress) (8, 61, 73, 110, 111, 125, 129, 134, 142), suggesting a cerebral effect across various brain areas. Importantly, it has been reported that hypnosedation was able to reduce both the affective and sensory components of pain (i.e., unpleasantness, intensity) compared to control conditions (33, 34, 36, 103, 105). Several studies focusing on brain electrical signals have highlighted changes in brain activity due to hypnosis-induced states (60, 62, 104, 149). Functional MRI (fMRI), positron emission tomography (PET), and laser-evoked potential (LEP) studies reported various changes in functional brain connectivity in participants during ongoing hypnosis (8). Interestingly, these changes mainly occurred in brain areas related to consciousness (e.g., anterior and posterior cingulate cortex, medial frontal cortex, precuneus) (8, 61, 140). Another study using hypnosis suggested that experimental pain will become less unpleasant even if the intensity stayed the same and was able to demonstrate the possibility to reduce brain imaging activity in regions related to affective components of pain (e.g., cingulate cortex and insula) without affecting the sensory-discriminative regions (e.g., somatosensory cortices) (103).

However, we must notice that there are some limits surrounding the clinical use of hypnosis. First, protocols using hypnosis frequently take a long time to administer to participants, and measurements are often limited to solely behavioral responses, without considering the subjective experience of the participants, especially to assess their hypnotizability (61, 141). This limit can lead to another constraint, which is that the number of participants often quite small (141, 143). In addition, methods can be subject to limitations, as some variations can occur across most studies, notably on the number of hypnosis sessions and intervention length or timing (especially regarding the induction phase), thus complicating interpretations and comparisons between the studies of this field (69). Although hypoalgesic benefits previously reported are promising, further studies will help expand these results in various acute and chronic pain conditions (Table 1).

### 2.2. Binaural beats

BBs can be defined as a perceived third frequency resulting from the difference between two different frequencies applied in each ear (e.g., a frequency of 253 Hz in the right ear and a 250 Hz one in the left ear will result in a third frequency of 3 Hz perceived in the brain) (19). Interestingly, it has been shown that this auditory stimulus can elicit an evoked synchronous response reproducing the same frequency and waveform of the stimulus entering the central auditory pathway as a brain oscillation (19). Thus, five different types of BBs have been listed, depending on their frequency: delta (i.e., 0.1–4 Hz), theta (i.e., 4–8 Hz), alpha (i.e., 8–13 Hz), beta (i.e., 13–30 Hz), and gamma (i.e., >30 Hz). To our knowledge, only theta- and alpha-BBs have been reported being able to elicit a hypoalgesic effect.

Theta-BB (i.e., 4–8 Hz) can significantly decrease the severity of perceived chronic pain compared to neutral sound (151). In this study, 36 patients suffering from different types of chronic pain had to listen to a 6 Hz BB tone for 20 min during 14 successive days, while a sham intervention group listened to a non-BB tone of 300 Hz. The results indicated reduced perceived pain severity exclusively in the theta-BB group. Although the pain scores were reduced in both groups, the authors observed a 77% larger reduction of the mean pain scores in favor of the theta-BB group (151).

Concerning alpha rhythms (i.e., 8–13 Hz), several studies indicate that a global suppression of alpha oscillations in somatosensory, motor, and visual areas are observed in response to both transient and tonic painful stimuli (98, 101). In some studies, pain intensity ratings were correlated with a decrease in alpha power (6, 87). More recently, a study has shown that listening to alpha-BB for 10 min significantly decreased the ratings of experimentally induced pain, compared to a control group (27). The authors also discovered that this effect was maximized for 10 Hz frequencies, compared to 8 and 12 Hz. However, the same study found no statistically significant differences with the control group for several aspects of the quality of life (e.g., anxiety, wellbeing, drowsiness), although these results contradict another study that found that alpha rhythm stimulation reduces both stress and anxiety (13). Interestingly, they also noted an improvement in heart rate variability via parasympathetic reinforcement, highlighting the ability of BBs to act on physiological variables as well. Thus, by reducing stress, anxiety, and physiological parameters, alpha rhythm stimulation could potentially decrease pain perception. In addition to their involvement in pain, a recent study pointed out the link between alpha rhythm and memory, by showing that listening to alpha-BB for 15 min can enhance memory recall (88).

However, it is important to notice that we found three studies that failed to promote brain oscillations following theta-, alpha-, beta-, or gamma-BBs, listening for durations of (i) 2 min (43), (ii) 3 min (75), or (iii) 5 min (39). Based on these findings, more studies are needed to investigate the potential hypoalgesic power of all types of BB in order to choose protocols adequately for brain oscillation-induced states (Table 1).

### 2.3. Colored noise

The “color” of a noise is a terminology used to classify different noises according to their power spectrum density (i.e., frequency of a sound), similar to light waves classification (26). For instance, if we decide to draw the sound wave diagram for “pink noise” by transposing it into a diagram of light waves, it would correspond to a pink light. As such, warm colors are assigned to low-frequency sounds, while cold colors are related to high-frequency sounds. To our knowledge, only white and pink noises have been related to pain analgesia studies (Table 1).

White noise has been extensively studied on sleep, mainly in infants and children (122), as well as intensive care unit patients (31, 123), although, at high intensity, it has been revealed in rodents that white noises can be harmful to the organism, creating anxiety-like behaviors as well as inducing apoptosis, chromatolysis, cytoplasmic organelle destruction, and glial activation brain structures (153). Concerning studies on pain, a team compared the effect of an MRI scanner noise to white noises on the sensory-discriminative (i.e., intensity, localization) and the motivational-affective (i.e., unpleasantness) components of pain (11). They showed that both the MRI scanner noise and white noises significantly reduced unpleasantness ratings, whereas the ability to locate pain was not significantly affected. Interestingly, they assume that, by acting solely on the motivational-affective component of pain, without affecting the sensory-discriminative one, noises may have therapeutic implications by diminishing the distress associated with the pain unpleasantness, while maintaining the capacity to localize the pain to avoid further injury. Another study demonstrated that the pain score was lowered with white noises in newborns (i.e., 38–42 weeks old) during an acute painful procedure (i.e., blood draw), compared to control conditions (67). Similar results were discovered to relieve procedural pain caused by vaccination in premature infants compared to a control group (71). Unfortunately, the impact of white noise in adults has mostly been studied regarding higher cognitive functions, such as semantic priming (4) or recognition memory tasks (107), and not pain.

Pink noise has been studied extensively in relation to sleep (12, 90, 93, 94, 152). A study recently investigated the effects of pink noise on pain in a rat model of chronic pain (12). They measured mechanical allodynia responses before and after exposure to pink noise and observed a statistically significant decrease in behavioral pain response in rats exposed to pink noise. It is important to notice that pink noise has also been used as a control stimulation, compared to music, in a study aiming to investigate the hypoalgesic effect of music on fibromyalgia pain (41). The results indicated a reduction in pain and an increased functional mobility in the music group, whereas there was no change in the pink noise control group. Consecutively, it is difficult to conclude the hypoalgesic power of pink noises.

To our knowledge, no scientific data have been published in order to highlight the potential hypoalgesic power on acute or chronic pain for other types of noise, including red, black, gray, green, blue, and purple noise (26). Concerning brown noise, we found only one study that has been published with stimulation close enough to brown noise, but relative to consciousness and not pain (115). They showed high stability of a Ganzfeld-induced state (i.e., altered state of consciousness through visual and auditory perceptual field homogenization following an unstructured sensory input), white noise displaying the highest overall global scores. Interestingly, the authors suggest that, based on their results, white noise could be very effective to homogenize the auditory field while ignoring potential environment distraction (115).

### 2.4. Bilateral alternative stimulation

Bilateral alternative stimulation (BAS) is a visual technique mainly applied during a psychotherapeutic approach called eye movement desensitization and reprocessing (EMDR) for post-traumatic stress disorder (PTSD) treatment (139, 147), although growing evidence tends to highlight possible applications of EMDR to relieve pain in patients living with acute or chronic painful conditions (44, 80, 130, 131, 42, 114).

We found a recent review of two RCTs related to acute pain and EMDR (114). The first one found that one session of EMDR was efficient to diminish acute experimental pain intensity (i.e., cold pain pressor), as well as to improve the pain threshold, bettering pain tolerance and reducing anxiety (48). The second one highlighted that one 60-min session 2 h post-abdominal surgery in an emergency service effectively decreased pain intensity (79).

A systematic review reported that EMDR has been efficient in decreasing pain intensity in several studies with patients suffering from various chronic pain conditions (e.g., phantom limb pain, headache, musculoskeletal pain, fibromyalgia) (130). An RCT pilot study also highlighted that EMDR effectively diminished pain intensity, sometimes for up to 6 months, in patients suffering from non-specific chronic back pain after 10 sessions of EMDR (42). In addition, nine weekly sessions of 1 h of EMDR can effectively decrease pain levels and their affect, as well as increase pain control in patients suffering from chronic pain for at least 6 months (44). In 38 patients living with chronic pain, it has been shown that 12 weekly EMDR sessions of 90 min were able to significantly decrease the amount of medication needed and to improve the quality of life (e.g., pain, physical activity, vitality, social interaction, emotional management) (80).

Little is known concerning the possible mechanisms through which EMDR might diminish pain. Pioneer EMDR protocols from Shapiro highlight eight phases to optimize the efficacy of the technique: (i) history and treatment plan, (ii) introduction to EMDR protocol and development of coping strategies, (iii) evaluation of the treatment targets, (iv) desensitization and reprocessing, (v) incorporation of positive cognitions, (vi) body scan (and the reprocessing of any remaining negative bodily sensations), (vii) relaxation (i.e., re-establishing emotional stability if distress has been experienced, and for use between sessions), and, finally, (viii) re-evaluation (116, 117, 114, 139). Interestingly, Grant and Threlfo specified that to facilitate relaxation and to change pain sensations, EMDR was usually accompanied by suggestions to ask the patient to shift their attention from the pain to the BAS (44). An fMRI study reported that BAS can either increase or decrease the activation of limbic structures (i.e., right amygdala, left dorsolateral prefrontal), thus highlighting the effect of EMDR on emotion processing in healthy participants (49). Interestingly, it seems that visual BAS are more effective than auditive BAS on memory tasks (57).

Finally, it is important to note that, despite an increased amount of evidence showing the benefits of EMDR on pain, studies usually do not use an active control group to address the results, and their monocentric design leads to small sample sizes (131). These results thus need to be extended in future investigations to confirm the potential hypoalgesic power of BAS (Table 1).

## 3. Sensory stimuli associated with virtual reality

In this review, we previously highlighted how VR or isolated sensory stimuli (i.e., hypnosis, binaural beats, colored noise, bilateral alternative stimulation) can elicit a hypoalgesic effect. This section aims to investigate the hypoalgesic power of combining these stimuli in a VR environment (Table 1).

### 3.1. Virtual reality and hypnosis

As hypnosis has been used for many decades, since the 21st century, its use in a 3D environment following the emergence of VR was rapidly tested in the early 2000s (78, 110, 111, 134). In 2010, Patterson et al. investigated how a hypnotic induction and hypoalgesic suggestions delivered by a customized VR hardware/software would be able to assess analgesia in patients with physical trauma at the hospital (96). They showed that pain intensity ratings, as well as pain unpleasantness, were significantly lowered in the group with VR and hypnosis compared to the groups with only VR or standard care alone, up to 8 hours post-treatment. Interestingly, a recent review and meta-analysis on hypnosis suggests that VR could potentiate the efficacy of hypnosis, especially in low hypnotic suggestibility patients (73). This observation is partially based on an RCT that highlighted that hypnosis with 3D VR animation can improve the user's mood, as well as reduce both the tiredness and the level of cortisol, measured through a salivary test (133). Meanwhile, it has recently been shown that hypnosis added with VR can effectively reduce pain, as well as anxiety and fatigue, in patients undergoing cardiac surgery (110, 111). However, it is important to notice that a study recently showed that human care was preferable to hypnosis through VR in patients undergoing electrophysiology and pacing procedures to improve their comfort (20).

Nevertheless, authors have highlighted some limits surrounding VR hypnosis induction, notably in case-series designs, such as an absence of a randomized distribution and a control group as well as possibly a small sample size (30). The methods can also be affected, since choosing the proper amount of VR sessions to induce hypnosis can often be limited across studies (110, 111). Further studies are needed to clarify all the possibilities of VR hypnotic induction on pain analgesia, but recent findings tend to encourage its use for alleviating patient's pain (14) (Table 1).

### 3.2. Virtual reality and binaural beats

To our knowledge, only a few studies recently investigated the potentializing effect of BB through VR (Table 1). In 2019, Perales et al. reported that some BBs (i.e., delta, theta, alpha) coupled with a VR environment can act on the sympathetic nervous system modalities (e.g., electrodermal activity) in healthy participants, in addition to other physiological parameters (e.g., temperature, heart rate) for children living with chronic pain (99, 100). The authors mention that these changes could introduce the user into an effective relaxation mood, potentially leading to an improvement in the perception of pain. More recently, they confirmed these results by showing a potentialized effect of VR with BB for chronic pain in children, but surprisingly not on the physiological modalities (i.e., heart rate, galvanic skin response), possibly due to study design limitations (109). Interestingly, it has recently been highlighted that BB in a VR environment can also drastically decrease the main aversive event of VR use, cybersickness, suggesting a potentially better efficacy of DTx using VR and BB (23).

### 3.3. Virtual reality and colored noise

As mentioned above, VR greatly benefits from immersion to generate its efficacy (18, 74). To our knowledge, studies that specifically investigated a potentialized effect of colored noise on VR analgesia are quite rare (Table 1), although we found one study that showed that adding sounds to a VR game can significantly increase pain tolerance for experimental thermal pain compared to the sounds or the VR game separately (64). However, the authors specified that “sounds” cited referred to the game's music, thus preventing us from concluding the specific hypoalgesic effect of white or pink noise when incorporated into a VR environment. Interestingly, it has recently been shown that shifting a music volume to the same frequency (i.e., 0.1 Hz) as the VR environment motion does not influence the body sway assessed by position measurements, suggesting a lack of effect of colored noise on cybersickness (24).

### 3.4. Virtual reality and bilateral alternative stimulation

To our knowledge, only one study has investigated more specifically the effect of transmitting EMDR techniques through a VR environment (Table 1). Kaminska et al. reported in 2020 that BAS in VR can significantly reduce the acute stress level as well as mood improvements in healthy adult volunteers, leading to be considered a great tool when added to a relaxation training program (66). Even if it is difficult to conclude with only one study, it seems that BAS through VR could benefit analgesia.

## 4. Conclusion

In this review, we highlighted (i) the hypoalgesic power of VR only, (ii) the hypoalgesic power of various sensory stimuli only (i.e., hypnosis, binaural beats, colored noise, bilateral alternative stimulation), and (iii) the potentialized hypoalgesic power of these sensory stimuli in a VR environment. In the first part, we have summarized many studies that showed with self-reported scales scores and cerebral imaging that VR can effectively reduce pain perception, both in healthy participants and during acute and chronic pain conditions, likely through the immersive capacity of VR. In addition, we found that the hypoalgesic effect of VR is sometimes as powerful as strong pharmacological analgesics (i.e., opioids). In the second part, we highlighted (i) how hypnosis can elicit an hypoalgesic effect as well as an improvement of the quality of life of participants, (ii) that some BB (i.e., theta, alpha) can effectively produce an hypoalgesic effect, likely by acting on cerebral oscillations, (iii) the low but existing hypoalgesic power of some colored noise (i.e., white, pink), and (iv) how an EMDR technique (i.e., BAS) may both decrease pain and improve the quality of life of some patients suffering from acute or chronic pain. In the final part, we reported the short but emerging scientific literature investigating the potentialized hypoalgesic effect of combining previous sensory stimuli evoked with a VR environment.

A potential limit to our review is the difficulty to compare all these modalities (i.e., VR only, stimuli only, VR and stimuli) as the methods are different across studies, even in the same fields of research. Moreover, the small sample size and the lack of information on the effect size in several studies are limiting a final conclusion on the clinical relevance of these studies. Another limit could be the non-exhaustivity of the sensory stimuli chosen in this review. Further reviews should investigate the hypoalgesic power of a plethora of other sensory stimuli (e.g., odors, textures, biofeedback) or cognitive approaches (e.g., cardiac coherence, mindfulness breathing) and their probable potentialized effect while being coupled with VR technologies. The fact that some studies report a hypoalgesic effect of the VR session outlasting hours and even months may be explainable by several mechanisms. For instance, the activation of endogenous pain modulation may outlast the effect by minutes or even hours. However, the longer effects may be explained by some life habit changes such as moving more freely and more frequently after the positive effect of VR. More studies are needed to characterize the different variables that may contribute to the long-term effect of VR.

We recently published an RCT (NCT04650516) where we highlighted that a VR treatment comprised of some sensory stimuli mentioned above (e.g., BB, BAS) effectively diminished the mean pain intensity in 45 patients diagnosed with moderate-to-severe endometriosis-related chronic pelvic pain, up to 4 h post-treatment, as well as reducing the mean perceived pain, compared to a 2D digital control (84). These results encourage us to conclude that VR with added sensory stimuli can be a good addition to an arsenal for alleviating pain. However, since the control was with the same 2D environment without the additional stimuli, we can only conclude the potential effects of the combination of these stimuli. Future studies are needed to better characterize the potentializing effect of adding BB, BAS, EMDR, or different sound frequencies on the hypoalgesic effect of VR.

In conclusion, our review suggests that adding sensory stimuli to VR can be a great opportunity for a plethora of DTx in order to alleviate patients from painful symptoms. It suggests that we can increase the efficacy of DTx analgesia with the addition of different sensory stimuli combined with VR.

## References

[1] Ahmadpour N Randall H Choksi H Gao A Vaughan C Poronnik P. Virtual reality interventions for acute and chronic pain management. Int J Biochem Cell Biol. (2019) 114:105568. 10/gg7h92 31306747 10.1016/j.biocel.2019.105568

[2] Al-Ghamdi NA Meyer WJ 3rd Atzori B Alhalabi W Seibel CC Ullman D Virtual reality analgesia with interactive eye tracking during brief thermal pain stimuli: a randomized controlled trial (crossover design). Front Hum Neurosci. (2020) 13:467. 10.3389/fnhum.2019.00467 32038200 PMC6990370

[3] Alrimy T Alhalabi W Malibari AA Alzahrani FS Alrajhi S Alhalabi M Virtual reality animal rescue world: pediatric virtual reality analgesia during just noticeable pressure pain in children aged 2–10 years old (crossover design). Front Psychol. (2022) 13:963765. 10.3389/fpsyg.2022.963765 36389517 PMC9651058

[4] Angwin AJ Wilson WJ Copland DA Barry RJ Myatt G Arnott WL. The impact of auditory white noise on semantic priming. Brain Lang. (2018) 180–182:1–7. 10.1016/j.bandl.2018.04.001 29653279

[5] Atzori B Vagnoli L Graziani D Hoffman HG Sampaio M Alhalabi W An exploratory study on the effectiveness of virtual reality analgesia for children and adolescents with kidney diseases undergoing venipuncture. Int J Environ Res Public Health. (2022) 19(4):2291. 10.3390/ijerph19042291 35206481 PMC8872518

[6] Babiloni C Brancucci A Percio CD Capotosto P Arendt-Nielsen L Chen ACN Anticipatory electroencephalography alpha rhythm predicts subjective perception of pain intensity. J Pain. (2006) 7(10):709–17. 10.1016/j.jpain.2006.03.005 17018331

[7] Bermo MS Patterson D Sharar SR Hoffman H Lewis DH. Virtual reality to relieve pain in burn patients undergoing imaging and treatment. Top Magn Reson Imaging (2020) 29(4):203–8. 10.1097/RMR.0000000000000248 32511197

[8] Bicego A Rousseaux F Faymonville ME Nyssen AS Vanhaudenhuyse A. Neurophysiology of hypnosis in chronic pain: a review of recent literature. Am J Clin Hypn. (2021) 64(1):62–80. 10.1080/00029157.2020.1869517 34748463

[9] Bordeleau M Stamenkovic A Tardif P-A Thomas J. The use of virtual reality in back pain rehabilitation: a systematic review and meta-analysis. J Pain. (2022) 23(2):175–95. 10.1016/j.jpain.2021.08.001 34425250

[10] Botella C Garcia-Palacios A Vizcaíno Y Herrero R Baños RM Belmonte MA. Virtual reality in the treatment of fibromyalgia: a pilot study. Cyberpsychol Behav Soc Netw. (2013) 16(3):215–23. 10.1089/cyber.2012.1572 23496678

[11] Boyle Y Bentley DE Watson A Jones AK. Acoustic noise in functional magnetic resonance imaging reduces pain unpleasantness ratings. NeuroImage. (2006) 31(3):1278–83. 10/fdbhhs 16517183 10.1016/j.neuroimage.2006.01.025

[12] Caravan B Hu L Veyg D Kulkarni P Zhang Q Chen ZS Sleep spindles as a diagnostic and therapeutic target for chronic pain. Mol Pain. (2020) 16:174480692090235. 10.1177/1744806920902350 PMC6977222 31912761

[13] Casciaro F Laterza V Conte S Pieralice M Federici A Todarello O Alpha-rhythm stimulation using brain entrainment enhances heart rate variability in subjects with reduced HRV. World J Neurosci. (2013) 03(04):213–20. 10.4236/wjns.2013.34028

[14] Célestin-Lhopiteau I Bioy A Bernard C. Chapitre 33. Réalité virtuelle thérapeutique et hypnose: Une médecine digitale à notre portée !, in Hypnoanalgésie et hypnosédation. Paris: Dunod (Aide-Mémoire) (2020). p. 256–61. 10.3917/dunod.celes.2020.01.0256

[15] Chan E Foster S Sambell R Leong P. (2018) Clinical efficacy of virtual reality for acute procedural pain management: a systematic review and meta-analysis. PLoS One. 13(7):e0200987. 10.1371/journal.pone.0200987 30052655 PMC6063420

[16] Chow H Hon J Chua W Chuan A. Effect of virtual reality therapy in reducing pain and anxiety for cancer-related medical procedures: a systematic narrative review. J Pain Symptom Manage. (2021) 61(2):384–94. 10/ghdvk8 32822755 10.1016/j.jpainsymman.2020.08.016

[17] Chuan A Zhou JJ Hou RM Stevens CJ Bogdanovych A. Virtual reality for acute and chronic pain management in adult patients: a narrative review. Anaesthesia. (2021) 76(5):695–704. 10.1111/anae.15202 32720308

[18] Colloca L Raghuraman N Wang Y Akintola T Brawn-Cinani B Colloca G Virtual reality: physiological and behavioral mechanisms to increase individual pain tolerance limits. Pain. (2020) 161(9):2010–21. 10.1097/j.pain.0000000000001900 32345915 PMC7584744

[19] Corona-González CE Alonso-Valerdi LM Ibarra-Zarate DI. Personalized theta and beta binaural beats for brain entrainment: an electroencephalographic analysis. Front Psychol. (2021) 12:764068. 10.3389/fpsyg.2021.764068 34867666 PMC8636003

[20] Coulibaly I Cardelli LS Duflos C Moulis L Mandoorah B Nicoleau J Virtual reality hypnosis in the electrophysiology lab: when human treatments are better than virtual ones. J Clin Med. (2022) 11(13):3913. 10.3390/jcm11133913 35807198 PMC9267480

[21] Dang A Arora D Rane P. Role of digital therapeutics and the changing future of healthcare. J Family Med Prim Care. (2020) 9(5):2207. 10.4103/jfmpc.jfmpc_105_20 32754475 PMC7380804

[22] Dang A Dang D Rane P. The expanding role of digital therapeutics in the post-COVID-19 era. Open COVID J. (2021) 1(1):32–7. 10.2174/2666958702101010032

[23] Bukhari AAS Deng X Huang J Liu K Durrani M Zaman MH. Evaluating binaural beats as a therapeutic tool for intervening visually-induced motion sickness in virtual reality environments. J Rehman Med Inst. (2022) 8(1):3–11. 10.52442/jrmi.v8i1.397

[24] Dent S Burger K Stevens S Smith BD Streepey JW. The effect of music on body sway when standing in a moving virtual environment. PLoS One. (2021) 16(9):e0258000. 10.1371/journal.pone.0258000 34582503 PMC8478188

[25] Ding L Hua H Zhu H Zhu S Lu J Zhao K Effects of virtual reality on relieving postoperative pain in surgical patients: a systematic review and meta-analysis. Int J Surg. (2020) 82:87–94. 10/gpdnvv 32882400 10.1016/j.ijsu.2020.08.033

[26] Doyle J.A. and Evans A.C. What colour is neural noise? arXiv:1806.03704 [q-bio] [Preprint]. (2018) Available at: http://arxiv.org/abs/1806.03704 (Accessed 9 March, 2020).

[27] Ecsy K Jones AKP Brown CA. Alpha-range visual and auditory stimulation reduces the perception of pain. Eur J Pain. (2017) 21(3):562–72. 10/f9sppf 27807916 10.1002/ejp.960

[28] Eijlers R Utens EM Staals LM de Nijs PF Berghmans JM Wijnen RM Systematic review and meta-analysis of virtual reality in pediatrics: effects on pain and anxiety. Anesth Analg. (2019) 129(5):1344–53. 10.1213/ANE.0000000000004165 31136330 PMC6791566

[29] Elkins GR Barabasz AF Council JR Spiegel D. Advancing research and practice: the revised APA division 30 definition of hypnosis. Am J Clin Hypn. (2015) 57(4):378–85. 10.1080/00029157.2015.1011465 25928776

[30] Enea V Dafinoiu I Opriş D David D. Effects of hypnotic analgesia and virtual reality on the reduction of experimental pain among high and low hypnotizables. Int J Clin Exp Hypn. (2014) 62(3):360–77. 10.1080/00207144.2014.901087 24837064

[31] Farokhnezhad Afshar P Bahramnezhad F Asgari P Shiri M. Effect of white noise on sleep in patients admitted to a coronary care. J Caring Sci. (2016) 5(2):103–9. 10.15171/jcs.2016.011 27354974 PMC4923834

[32] Faymonville ME Fissette J Mambourg PH Roediger L Joris J Lamy M. Hypnosis as adjunct therapy in conscious sedation for plastic surgery. Reg Anesth. (1995) 20(2):145–51. PMID: . 7605762

[33] Faymonville EM Mambourg HP Joris J Vrijens B Fissette J Psychological approaches during conscious sedation. Hypnosis versus stress reducing strategies: a prospective randomized study. Pain. (1997) 73(3):361–7. 10/fsm5cc 9469526 10.1016/S0304-3959(97)00122-X

[34] Faymonville ME Roediger L Del Fiore G Delgueldre C Phillips C Lamy M Increased cerebral functional connectivity underlying the antinociceptive effects of hypnosis. Brain Res Cogn Brain Res. (2003) 17(2):255–62. 10/b3mqnx 12880897 10.1016/s0926-6410(03)00113-7

[35] Faymonville M-E Boly M Laureys S. Functional neuroanatomy of the hypnotic state. J Physiol Paris. (2006) 99(4–6):463–9. 10/cpw7v4 16750615 10.1016/j.jphysparis.2006.03.018

[36] Faymonville ME Fiore GD Maquet P. Neural mechanisms of antinociceptive effects of hypnosis. Anesthesiology. (2000) 92(5):11. 10.1097/00000542-200005000-00013 10781270

[37] Fletcher D Martinez V. Opioid-induced hyperalgesia in patients after surgery: a systematic review and a meta-analysis. Br J Anaesth. (2014) 112(6):991–1004. 10/f545rw 24829420 10.1093/bja/aeu137

[38] Frey DP Bauer ME Bell CL Low LK Hassett AL Cassidy RB Virtual reality analgesia in labor: the VRAIL pilot study—a preliminary randomized controlled trial suggesting benefit of immersive virtual reality analgesia in unmedicated laboring women. Anesth Analg. (2019) 128(6):e93–6. 10/ggmc7g 31094789 10.1213/ANE.0000000000003649

[39] Gao X Cao H Ming D Qi H Wang X Wang X Analysis of EEG activity in response to binaural beats with different frequencies. Int J Psychophysiol. (2014) 94(3):399–406. 10/f6whqg 25448376 10.1016/j.ijpsycho.2014.10.010

[40] Garcia LM Birckhead BJ Krishnamurthy P Sackman J Mackey IG Louis RG An 8-week self-administered at-home behavioral skills-based virtual reality program for chronic low back pain: double-blind, randomized, placebo-controlled trial conducted during COVID-19. J Med Internet Res. (2021) 23(2):e26292. 10/gngz7m 33484240 10.2196/26292 PMC7939946

[41] Garza-Villarreal EA Wilson AD Vase L Brattico E Barrios FA Jensen TS Music reduces pain and increases functional mobility in fibromyalgia. Front Psychol. (2014) 5:90. 10.3389/fpsyg.2014.00090 24575066 PMC3920463

[42] Gerhardt A Leisner S Hartmann M Janke S Seidler GH Eich W Eye movement desensitization and reprocessing vs. Treatment-as-usual for non-specific chronic back pain patients with psychological trauma: a randomized controlled pilot study. Front Psychiatry. (2016) 7:201. Available at: https://www.frontiersin.org/article/10.3389/fpsyt.2016.00201 (Accessed: 1 February, 2022). 10.3389/fpsyt.2016.00201 28066274 PMC5167699

[43] Goodin P Ciorciari J Baker K Carey AM Harper M Kaufman J. A high-density EEG investigation into steady state binaural beat stimulation. PLoS One. (2012) 7(4):e34789. 10.1371/journal.pone.0034789 22496862 PMC3322125

[44] Grant M Threlfo C. EMDR in the treatment of chronic pain. J Clin Psychol. (2002) 58(12):1505–20. 10/frhrsx 12455018 10.1002/jclp.10101

[45] Guo C Deng H Yang J. Effect of virtual reality distraction on pain among patients with hand injury undergoing dressing change. J Clin Nurs. (2015) 24(1–2):115–20. 10/f63zq3 24899241 10.1111/jocn.12626

[46] Gutiérrez-Maldonado J Gutiérrez-Martínez O Loreto-Quijada D Nieto-Luna R. The use of virtual reality for coping with pain with healthy participants. Psicothema. (2012) 24(4):516–22. PMID: . 23079345

[47] Hadjiat Y Marchand S. Virtual reality and the mediation of acute and chronic pain in adult and pediatric populations: research developments. Front Pain Res. (2022) 3:840921. 10.3389/fpain.2022.840921 PMC9120608 35599969

[48] Hekmat H Groth S Rogers D. Pain ameliorating effect of eye movement desensitization. J Behav Ther Exp Psychiatry. (1994) 25(2):121–9. 10/b5c7pk 7983221 10.1016/0005-7916(94)90004-3

[49] Herkt D Tumani V Grön G Kammer T Hofmann A Abler B. Facilitating access to emotions: neural signature of EMDR stimulation. PLoS One. (2014) 9(8):e106350. 10.1371/journal.pone.0106350 25165974 PMC4148424

[50] Hoffman HG Doctor JN Patterson DR Carrougher GJ Furness TA 3rd. Virtual reality as an adjunctive pain control during burn wound care in adolescent patients. Pain. (2000) 85(1–2):305–9. 10.1016/s0304-3959(99)00275-4 10692634

[51] Hoffman HG Richards TL Coda B Bills AR Blough D Richards AL Modulation of thermal pain-related brain activity with virtual reality: evidence from fMRI. NeuroReport. (2004) 15(8):1245–8. 10/d6q43p 15167542 10.1097/01.wnr.0000127826.73576.91

[52] Hoffman HG Seibel EJ Richards TL Furness TA Patterson DR Sharar SR. Virtual reality helmet display quality influences the magnitude of virtual reality analgesia. J Pain. (2006) 7(11):843–50. 10/dkndds 17074626 10.1016/j.jpain.2006.04.006

[53] Hoffman HG Richards TL Van Oostrom T Coda BA Jensen MP Blough DK The analgesic effects of opioids and immersive virtual reality distraction: evidence from subjective and functional brain imaging assessments. Anesth Analg. (2007) 105(6):1776–83; table of contents. 10.1213/01.ane.0000270205.45146.db 18042882

[54] Hoffman HG Chambers GT Meyer WJ 3rd Arceneaux LL Russell WJ Seibel EJ Virtual reality as an adjunctive non-pharmacologic analgesic for acute burn pain during medical procedures. Ann Behav Med. (2011) 41(2):183–91. 10/fjwzqq 21264690 10.1007/s12160-010-9248-7 PMC4465767

[55] Hoffmann J May A. Diagnosis, pathophysiology, and management of cluster headache. Lancet Neurol. (2018) 17(1):75–83. 10.1016/S1474-4422(17)30405-2 29174963

[56] Honzel E Murthi S Brawn-Cinani B Colloca G Kier C Varshney A Virtual reality, music, and pain: developing the premise for an interdisciplinary approach to pain management. Pain. (2019) 160(9):1909–19. 10.1097/j.pain.0000000000001539 30817437 PMC7279616

[57] van den Hout MA Engelhard IM Rijkeboer MM Koekebakker J Hornsveld H Leer A EMDR: eye movements superior to beeps in taxing working memory and reducing vividness of recollections. Behav Res Ther. (2011) 49(2):92–8. 10/b28mgt 21147478 10.1016/j.brat.2010.11.003

[58] Indovina P Barone D Gallo L Chirico A De Pietro G Giordano A. Virtual reality as a distraction intervention to relieve pain and distress during medical procedures: a comprehensive literature review. Clin J Pain. (2018) 34(9):858–77. 10.1097/AJP.0000000000000599 29485536

[59] JahaniShoorab N Ebrahimzadeh Zagami S Nahvi A Mazluom SR Golmakani N Talebi M The effect of virtual reality on pain in primiparity women during episiotomy repair: a randomize clinical trial. Iran J Med Sci. (2015) 40(3):6. PMID: . 25999621 PMC4430883

[60] Jensen MP Sherlin LH Gertz KJ Braden AL Kupper AE Gianas A Brain EEG activity correlates of chronic pain in persons with spinal cord injury: clinical implications. Spinal Cord. (2013) 51(1):55–8. 10.1038/sc.2012.84 22801188

[61] Jensen MP Jamieson GA Lutz A Mazzoni G McGeown WJ Santarcangelo EL New directions in hypnosis research: strategies for advancing the cognitive and clinical neuroscience of hypnosis. Neurosci Conscious. (2017) 2017:1. 10.1093/nc/nix004 PMC5635845 29034102

[62] Jensen MP Day MA Miró J. Neuromodulatory treatments for chronic pain: efficacy and mechanisms. Nat Rev Neurol. (2014) 10(3):167–78. 10/f5vm6w 24535464 10.1038/nrneurol.2014.12 PMC5652321

[63] Jin W Choo A Gromala D Shaw C Squire P. A virtual reality game for chronic pain management: a randomized, controlled clinical study. Stud Health Technol Inform. (2016) 220:154–60. PMID: . 27046570

[64] Johnson S Coxon M. Sound can enhance the analgesic effect of virtual reality. R Soc Open Sci. (2016) 3(3):150567. 10.1098/rsos.150567 27069646 PMC4821257

[65] Jones T Moore T Choo J. The impact of virtual reality on chronic pain. PLoS One. (2016) 11:12. 10.1371/journal.pone.0167523 PMC5172565 27997539

[66] Kaminska D Smolka K Zwolinski G Wiak S Merecz-Kot D Anbarjafari G. Stress reduction using bilateral stimulation in virtual reality. IEEE Access. (2020) 8:200351–66. 10.1109/ACCESS.2020.3035540

[67] Karakoç A Türker F. Effects of white noise and holding on pain perception in newborns. Pain Manag Nurs. (2014) 15(4):864–70. 10.1016/j.pmn.2014.01.002 24559599

[68] Käthner I Bader T Pauli P. Heat pain modulation with virtual water during a virtual hand illusion. Sci Rep. (2019) 9(1):1–12. 10/gg7rq4 31836829 10.1038/s41598-019-55407-0 PMC6911006

[69] Kendrick C Sliwinski J Yu Y Johnson A Fisher W Kekecs Z Hypnosis for acute procedural pain: a critical review. Int J Clin Exp Hypn. (2016) 64(1):75–115. 10.1080/00207144.2015.1099405 26599994 PMC5120961

[70] Kiryu T So RH. Sensation of presence and cybersickness in applications of virtual reality for advanced rehabilitation. J Neuroeng Rehabil. (2007) 4:34. 10.1186/1743-0003-4-34 17894857 PMC2117018

[71] Kucukoglu S Aytekin A Celebioglu A Celebi A Caner I Maden R. Effect of white noise in relieving vaccination pain in premature infants. Pain Manag Nurs. (2016) 17(6):392–400. 10.1016/j.pmn.2016.08.006 27751753

[72] Lambert V Boylan P Boran L Hicks P Kirubakaran R Devane D Virtual reality distraction for acute pain in children. Cochrane Database Syst Rev. (2020) 10(10):CD010686. 10.1002/14651858.CD010686.pub2 33089901 PMC8094164

[73] Langlois P Perrochon A David R Rainville P Wood C Vanhaudenhuyse A Hypnosis to manage musculoskeletal and neuropathic chronic pain: a systematic review and meta-analysis. Neurosci Biobehav Rev. (2022) 135:104591. 10.1016/j.neubiorev.2022.104591 35192910

[74] Lewis GN Rosie JA. Virtual reality games for movement rehabilitation in neurological conditions: how do we meet the needs and expectations of the users? Disabil Rehabil. (2012) 34(22):1880–6. 10.3109/09638288.2012.670036 22480353

[75] López-Caballero F Escera C. Binaural beat: a failure to enhance EEG power and emotional arousal. Front Hum Neurosci. (2017) 11:557. 10.3389/fnhum.2017.00557 29187819 PMC5694826

[76] López-Valverde N Muriel-Fernández J López-Valverde A Valero-Juan LF Ramírez JM Flores-Fraile J Use of virtual reality for the management of anxiety and pain in dental treatments: systematic review and meta-analysis. J Clin Med. (2020) 9(10):3086. 10.3390/jcm9103086 32987885 PMC7600113

[77] Maddern J Grundy L Castro J Brierley SM. Pain in endometriosis. Front Cell Neurosci. (2020) 14:590823. 10/gpc2g2 33132854 10.3389/fncel.2020.590823 PMC7573391

[78] Mallari B Spaeth EK Goh H Boyd BS. Virtual reality as an analgesic for acute and chronic pain in adults: a systematic review and meta-analysis. J Pain Res. (2019) 12:2053–85. 10/gg7rtr 31308733 10.2147/JPR.S200498 PMC6613199

[79] Maroufi M Zamani S Izadikhah Z Marofi M O'Connor P. Investigating the effect of eye movement desensitization and reprocessing (EMDR) on postoperative pain intensity in adolescents undergoing surgery: a randomized controlled trial. J Adv Nurs. (2016) 72(9):2207–17. 10.1111/jan.12985 27134066

[80] Mazzola A Calcagno ML Goicochea MT Pueyrredòn H Leston J Salvat F. L’EMDR dans le traitement de la douleur chronique. J EMDR Pract Res. (2010) 4(3):31–44. 10/dnrqwd

[81] McCauley ME Sharkey TJ. Cybersickness: perception of self-motion in virtual environments. Presence. (1992) 1(3):311–8. 10.1162/pres.1992.1.3.311

[82] McSherry T Atterbury M Gartner S Helmold E Searles DM Schulman C. Randomized, crossover study of immersive virtual reality to decrease opioid use during painful wound care procedures in adults. J Burn Care Res. (2017) 1:278–85. 10.1097/BCR.0000000000000589 28570305

[83] Melo M Goncalves G Monteiro P Coelho H Vasconcelos-Raposo J Bessa M. Do multisensory stimuli benefit the virtual reality experience? A systematic review. IEEE Trans Visualiz Comp Graph. (2022) 28(2):1428–42. 10.1109/TVCG.2020.3010088 32746276

[84] Merlot B Dispersyn G Husson Z Chanavaz-Lacheray I Dennis T Greco-Vuilloud J Pain reduction with an immersive digital therapeutic tool in women living with endometriosis-related pelvic pain: randomized controlled trial. J Med Internet Res. (2022) 24(9):e39531. 10.2196/39531 36129733 PMC9536521

[85] Mills SEE Nicolson KP Smith BH. Chronic pain: a review of its epidemiology and associated factors in population-based studies. Br J Anaesth. (2019) 123(2):e273–83. 10.1016/j.bja.2019.03.023 31079836 PMC6676152

[86] Mosso-Vázquez JL Gao K Wiederhold BK Wiederhold MD. Virtual reality for pain management in cardiac surgery. Cyberpsychol Behav Soc Netw. (2014) 17(6):371–8. 10/f56c9w 24892200 10.1089/cyber.2014.0198 PMC4043366

[87] Mouraux A Guérit JM Plaghki L. Non-phase locked electroencephalogram (EEG) responses to CO2 laser skin stimulations may reflect central interactions between A∂- and C-fibre afferent volleys. Clin Neurophysiol. (2003) 114(4):710–22. 10.1016/S1388-2457(03)00027-0 12686279

[88] Mujib MD Hasan MA Qazi SA Vuckovic A. Understanding the neurological mechanism involved in enhanced memory recall task following binaural beat: a pilot study. Exp Brain Res. (2021) 239(9):2741–54. 10/gpj89m 34232346 10.1007/s00221-021-06132-6 PMC8448692

[89] Nadeau SE Wu JK Lawhern RA. Opioids and chronic pain: an analytic review of the clinical evidence. Front Pain Res. (2021) 2:721357. 10.3389/fpain.2021.721357 PMC8915556 35295493

[90] Ngo HV Claussen JC Born J Mölle M. Induction of slow oscillations by rhythmic acoustic stimulation. J Sleep Res. (2013) 22(1):22–31. 10.1111/j.1365-2869.2012.01039.x 22913273

[91] Nicholson B Verma S. Comorbidities in chronic neuropathic pain. Pain Med. (2004) 5(suppl 1):S9–27. 10.1111/j.1526-4637.2004.04019.x 14996227

[92] Ortiz-Catalan M Guðmundsdóttir RA Kristoffersen MB Zepeda-Echavarria A Caine-Winterberger K Kulbacka-Ortiz K Phantom motor execution facilitated by machine learning and augmented reality as treatment for phantom limb pain: a single group, clinical trial in patients with chronic intractable phantom limb pain. Lancet. (2016) 388(10062):2885–94. 10.1016/S0140-6736(16)31598-7 27916234

[93] Papalambros NA Santostasi G Malkani RG Braun R Weintraub S Paller KA Acoustic enhancement of sleep slow oscillations and concomitant memory improvement in older adults. Front Hum Neurosci. (2017) 11:109. 10/ggsw9k 28337134 10.3389/fnhum.2017.00109 PMC5340797

[94] Papalambros NA Weintraub S Chen T Grimaldi D Santostasi G Paller KA Acoustic enhancement of sleep slow oscillations in mild cognitive impairment. Ann Clin Transl Neurol. (2019) 6(7):1191–201. 10.1002/acn3.796 31353857 PMC6649400

[95] Park MJ Kim DJ Lee U Na EJ Jeon HJ. A literature overview of virtual reality (VR) in treatment of psychiatric disorders: recent advances and limitations. Front Psychiatry. (2019) 10:505. 10.3389/fpsyt.2019.00505 31379623 PMC6659125

[96] Patterson DR Jensen MP Wiechman SA Sharar SR. Virtual reality hypnosis for pain associated with recovery from physical trauma. Int J Clin Exp Hypn. (2010) 58(3):288–300. 10/chxv3d 20509069 10.1080/00207141003760595 PMC2913598

[97] Pekyavas NO Ergun N. Comparison of virtual reality exergaming and home exercise programs in patients with subacromial impingement syndrome and scapular dyskinesis: short term effect. Acta Orthop Traumatol Turc. (2017) 51(3):238–42. 10.1016/j.aott.2017.03.008 28446376 PMC6197467

[98] Peng W Hu L Zhang Z Hu Y. Changes of spontaneous oscillatory activity to tonic heat pain. PLoS One. (2014) 9(3):e91052. 10.1371/journal.pone.0091052 24603703 PMC3946288

[99] Perales FJ Riera L Ramis S Guerrero A. Evaluation of a VR system for pain management using binaural acoustic stimulation. Multimed Tools Appl. (2019) 78(23):32869–90. 10.1007/s11042-019-07953-y

[100] Perales FJ Sanchez M Ramis S. A mood modulation using virtual reality and binaural sounds. Proceedings of the XX international conference on human computer interaction—interacción ‘19. The XX international conference, June 2019. Donostia, Spain. New York, NY: Association for Computing Machinery (2019). pp. 1–2. 10/gg7rhd

[101] Ploner M Gross J Timmermann L Pollok B Schnitzler A. Pain suppresses spontaneous brain rhythms. Cerebral Cortex. (2006) 16(4):537–40. 10.1093/cercor/bhj001 16033927

[102] Pourmand A Davis S Marchak A Whiteside T Sikka N. Virtual reality as a clinical tool for pain management. Curr Pain Headache Rep. (2018) 22(8):53. 10/gdr7hs 29904806 10.1007/s11916-018-0708-2

[103] Rainville P Duncan GH Price DD Carrier B Bushnell MC. Pain affect encoded in human anterior cingulate but not somatosensory cortex. Science. (1997) 277(5328):968–71. 10.1126/science.277.5328.968 9252330

[104] Rainville P Hofbauer RK Paus T Duncan GH Bushnell MC Price DD. Cerebral mechanisms of hypnotic induction and suggestion. J Cogn Neurosci. (1999) 11(1):110–25. 10/bfbkzg 9950718 10.1162/089892999563175

[105] Rainville P. Hypnosis and the analgesic effect of suggestions. Pain. (2008) 134(1):1–2. 10.1016/j.pain.2007.10.030 18037243

[106] Raja SN Carr DB Cohen M Finnerup NB Flor H Gibson S The revised international association for the study of pain definition of pain: concepts, challenges, and compromises. Pain. (2020) 161(9):1976–82. 10.1097/j.pain.0000000000001939 32694387 PMC7680716

[107] Rausch VH Bauch EM Bunzeck N. White noise improves learning by modulating activity in dopaminergic midbrain regions and right superior temporal sulcus. J Cogn Neurosci. (2014) 26(7):1469–80. 10/gg7rtd 24345178 10.1162/jocn_a_00537

[108] Rauschecker JP May ES Maudoux A Ploner M. Frontostriatal gating of tinnitus and chronic pain. Trends Cogn Sci (Regul Ed). (2015) 19(10):567–78. 10.1016/j.tics.2015.08.002 PMC4587397 26412095

[109] Riera L Verger S Montoya PJ Perales FJ. Advances in the cognitive management of chronic pain in children through the use of virtual reality combined with binaural beats: a pilot study. Adv Hum Comput Interact. (2022) 2022:2495182. 10.1155/2022/2495182

[110] Rousseaux F Faymonville ME Nyssen AS Dardenne N Ledoux D Massion PB Can hypnosis and virtual reality reduce anxiety, pain and fatigue among patients who undergo cardiac surgery: a randomised controlled trial. Trials. (2020) 21(1):330. 10.1186/s13063-020-4222-6 32293517 PMC7157998

[111] Rousseaux F Bicego A Ledoux D Massion P Nyssen AS Faymonville ME Hypnosis associated with 3D immersive virtual reality technology in the management of pain: a review of the literature. J Pain Res. (2020) 13:1129–38. 10.2147/JPR.S231737 32547176 PMC7247604

[112] Salsitz EA. Chronic pain, chronic opioid addiction: a complex nexus. J Med Toxicol. (2016) 12(1):54–7. 10/gn46xm 26602212 10.1007/s13181-015-0521-9 PMC4781803

[113] Sarig Bahat H Takasaki H Chen X Bet-Or Y Treleaven J. Cervical kinematic training with and without interactive VR training for chronic neck pain—a randomized clinical trial. Man Ther. (2015) 20(1):68–78. 10.1016/j.math.2014.06.008 25066503

[114] Scelles C Bulnes LC. EMDR as treatment option for conditions other than PTSD: a systematic review. Front Psychol. (2021) 12:644369. 10.3389/fpsyg.2021.644369 34616328 PMC8488430

[115] Schmidt TT Prein JC. The Ganzfeld experience—a stably inducible altered state of consciousness: effects of different auditory homogenizations. Psych J. (2019) 8(1):66–81. 10.1002/pchj.262 30609322

[116] Shapiro F. Eye movement desensitization: a new treatment for post-traumatic stress disorder. J Behav Ther Exp Psychiatry. (1989) 20(3):211–7. 10/cbthcv 2576656 10.1016/0005-7916(89)90025-6

[117] Shapiro F. EMDR, adaptive information processing, and case conceptualization. J EMDR Pract Res. (2007) 1(2):68–87. 10.1891/1933-3196.1.2.68

[118] Sharar SR Carrougher GJ Nakamura D Hoffman HG Blough DK Patterson DR. Factors influencing the efficacy of virtual reality distraction analgesia during postburn physical therapy: preliminary results from 3 ongoing studies. Arch Phys Med Rehabil. (2007) 88(12):S43–9. 10.1016/j.apmr.2007.09.004 18036981

[119] Sharar SR Alamdari A Hoffer C Hoffman HG Jensen MP Patterson DR. Circumplex model of affect: a measure of pleasure and arousal during virtual reality distraction analgesia. Games Health J. (2016) 5(3):197–202. 10.1089/g4h.2015.0046 27171578 PMC4931759

[120] Shiri S Feintuch U Weiss N Pustilnik A Geffen T Kay B A virtual reality system combined with biofeedback for treating pediatric chronic headache—a pilot study. Pain Med. (2013) 14(5):621–7. 10.1111/pme.12083 23659372

[121] Smith A Carlow K Biddulph T Murray B Paton M Harvie DS. Contextual modulation of pain sensitivity utilising virtual environments. Br J Pain. (2017) 11(2):71–80. 10.1177/2049463717698349 28491299 PMC5405974

[122] Spencer JA Moran DJ Lee A Talbert D. White noise and sleep induction. Arch Dis Child. (1990) 65(1):135–7. 10.1136/adc.65.1.135 2405784 PMC1792397

[123] Stanchina ML Abu-Hijleh M Chaudhry BK Carlisle CC Millman RP. The influence of white noise on sleep in subjects exposed to ICU noise. Sleep Med. (2005) 6(5):423–8. 10.1016/j.sleep.2004.12.004 16139772

[124] Staud R. Abnormal endogenous pain modulation is a shared characteristic of many chronic pain conditions. Expert Rev Neurother. (2012) 12(5):577–85. 10.1586/ern.12.41 22550986 PMC3373184

[125] Stoelb BL Molton IR Jensen MP Patterson DR. The efficacy of hypnotic analgesia in adults: a review of the literature. Contemp Hypn. (2009) 26(1):24–39. 10.1002/ch.370 20161034 PMC2753288

[126] Sweta V Abhinav R Ramesh A. Role of virtual reality in pain perception of patients following the administration of local anesthesia. Ann Maxillofac Surg. (2019) 9(1):110. 10.4103/ams.ams_263_18 31293937 PMC6585215

[127] Tan G Rintala DH Jensen MP Fukui T Smith D Williams W. A randomized controlled trial of hypnosis compared with biofeedback for adults with chronic low back pain. Eur J Pain. (2015) 19(2):271–80. 10.1002/ejp.545 24934738

[128] Tarrant J Viczko J Cope H. Virtual reality for anxiety reduction demonstrated by quantitative EEG: a pilot study. Front Psychol. (2018) 9:1280. 10.3389/fpsyg.2018.01280 30087642 PMC6066724

[129] Tefikow S Barth J Maichrowitz S Beelmann A Strauss B Rosendahl J. Efficacy of hypnosis in adults undergoing surgery or medical procedures: a meta-analysis of randomized controlled trials. Clin Psychol Rev. (2013) 33(5):623–36. 10.1016/j.cpr.2013.03.005 23628907

[130] Tesarz J Leisner S Gerhardt A Janke S Seidler GH Eich W Effects of eye movement desensitization and reprocessing (EMDR) treatment in chronic pain patients: a systematic review: systematic review: EMDR in chronic pain. Pain Med. (2014) 15(2):247–63. 10/f6sb9q 24308821 10.1111/pme.12303

[131] Tesarz J Wicking M Bernardy K Seidler GH. EMDR therapy's efficacy in the treatment of pain. J EMDR Pract Res. (2019) 13(4):337–44. 10.1891/1933-3196.13.4.337

[132] Theingi S Leopold I Ola T Cohen GS Maresky HS. Virtual reality as a non-pharmacological adjunct to reduce the use of analgesics in hospitals. J Cogn Enhanc. (2022) 6(1):108–13. 10.1007/s41465-021-00212-9 33842827 PMC8022314

[133] Thompson T Steffert T Steed A Gruzelier J. A randomized controlled trial of the effects of hypnosis with 3-D virtual reality animation on tiredness, mood, and salivary cortisol. Int J Clin Exp Hypn. (2010) 59(1):122–42. 10.1080/00207144.2011.522917 21104488

[134] Thompson T Terhune DB Oram C Sharangparni J Rouf R Solmi M The effectiveness of hypnosis for pain relief: a systematic review and meta-analysis of 85 controlled experimental trials. Neurosci Biobehav Rev. (2019) 99:298–310. 10.1016/j.neubiorev.2019.02.013 30790634

[135] de Tommaso M Ricci K Laneve L Savino N Antonaci V Livrea P. Virtual visual effect of hospital waiting room on pain modulation in healthy subjects and patients with chronic migraine. Pain Res Treat. (2013) 2013:1–8. 10.1155/2013/515730 PMC3556890 23365736

[136] Treede RD Rief W Barke A Aziz Q Bennett MI Benoliel R A classification of chronic pain for ICD-11. Pain. (2015) 156(6):1003–7. 10/f3ph69 25844555 10.1097/j.pain.0000000000000160 PMC4450869

[137] Treede RD Rief W Barke A Aziz Q Bennett MI Benoliel R Chronic pain as a symptom or a disease: the IASP classification of chronic pain for the international classification of diseases (ICD-11). Pain. (2019) 160(1):19–27. 10/gfvp3z 30586067 10.1097/j.pain.0000000000001384

[138] Understanding DTx. Digital therapeutics definition and core principles (2019). Available at: https://dtxalliance.org/understanding-dtx/ (Accessed January 2023).

[139] Valiente-Gómez A Moreno-Alcázar A Treen D Cedrón C Colom F Pérez V EMDR beyond PTSD: a systematic literature review. Front Psychol. (2017) 8:1668. 10/gb2zwx 29018388 10.3389/fpsyg.2017.01668 PMC5623122

[140] Vanhaudenhuyse A Demertzi A Schabus M Noirhomme Q Bredart S Boly M Two distinct neuronal networks mediate the awareness of environment and of self. J Cogn Neurosci. (2011) 23(3):570–8. 10.1162/jocn.2010.21488 20515407

[141] Vanhaudenhuyse A Ledoux D Gosseries O Demertzi A Laureys S Faymonville M-E. Can subjective ratings of absorption, dissociation, and time perception during “neutral hypnosis” predict hypnotizability?: an exploratory study. Int J Clin Exp Hypn. (2019) 67(1):28–38. 10.1080/00207144.2019.1553765 30702397

[142] Vanhaudenhuyse A Laureys S Faymonville M-E. Neurophysiology of hypnosis. Neurophysiol Clin. (2014) 44(4):343–53. 10.1016/j.neucli.2013.09.006 25306075

[143] Vanhaudenhuyse A Nyssen A-S Faymonville M-E. Recent insight on how the neuroscientific approach helps clinicians. OBM Integr Complement Med. (2020) 5(2):1–20. 10.21926/obm.icm.2002028

[144] Veličković P Milovanović M. Improvement of the interaction model aimed to reduce the negative effects of cybersickness in VR rehab applications. Sensors (Basel). (2021) 21(2):321. 10.3390/s21020321 33418838 PMC7824908

[145] Walker MR Kallingal GJ Musser JE Folen R Stetz MC Clark JY. Treatment efficacy of virtual reality distraction in the reduction of pain and anxiety during cystoscopy. Mil Med. (2014) 179(8):891–6. 10/f6d4fm 25102532 10.7205/MILMED-D-13-00343

[146] Weech S Kenny S Barnett-Cowan M. Presence and cybersickness in virtual reality are negatively related: a review. Front Psychol. (2019) 10:158. 10.3389/fpsyg.2019.00158 30778320 PMC6369189

[147] Welch KL Beere DB. Eye movement desensitization and reprocessing: a treatment efficacy model. Clin Psychol Psychother. (2002) 9(3):165–76. 10/c2qqjv

[148] Wiederhold BK Gao K Sulea C Wiederhold MD. Virtual reality as a distraction technique in chronic pain patients. Cyberpsychol Behav Soc Netw. (2014) 17(6):346–52. 10/f56fv9 24892196 10.1089/cyber.2014.0207 PMC4043365

[149] Williams JD Gruzelier JH. Differentiation of hypnosis and relaxation by analysis of narrow band theta and alpha frequencies. Int J Clin Exp Hypn. (2001) 49(3):185–206. 10.1080/00207140108410070 11430154

[150] Yang JH Ryu JJ Nam E Lee HS Lee JK. Effects of preoperative virtual reality magnetic resonance imaging on preoperative anxiety in patients undergoing arthroscopic knee surgery: a randomized controlled study. Arthroscopy. (2019) 35(8):2394–9. 10.1016/j.arthro.2019.02.037 31395176

[151] Zampi DD. Efficacy of theta binaural beats for the treatment of chronic pain. Altern Ther Health Med. (2016) 22(1):32–8. PMID: . 26773319

[152] Zhou J Liu D Li X Ma J Zhang J Fang J. Pink noise: effect on complexity synchronization of brain activity and sleep consolidation. J Theor Biol. (2012) 306:68–72. 10.1016/j.jtbi.2012.04.006 22726808

[153] Zhvania M Gogokhia N Tizabi Y Japaridze N Pochkidze N Lomidze N Behavioral and neuroanatomical effects on exposure to white noise in rats. Neurosci Lett. (2020) 728:134898. 10.1016/j.neulet.2020.134898 32224224

